# Plasma biomarkers of inflammation associate with blood pressure and arterial stiffness in adolescents after very preterm birth

**DOI:** 10.1093/cvr/cvae192

**Published:** 2024-08-28

**Authors:** Anja Meissner, Jonas Liefke, Frank Matthes, Eva Morsing, David Ley, Erik Hedström

**Affiliations:** Department of Experimental Medical Science, Lund University, Klinikgatan 32, SE-22184 Lund, Sweden; Wallenberg Centre for Molecular Medicine, Lund University, Klinikgatan 32, SE-22184 Lund, Sweden; Department of Physiology, Institute of Theoretical Medicine, Faculty of Medicine, University of Augsburg, Universitätsstr. 2, DE-86159 Augsburg, Germany; Clinical Physiology, Department of Clinical Sciences Lund, Lund University, Lund, Sweden; Department of Clinical Physiology, Skåne University Hospital, Lund, Sweden; Department of Experimental Medical Science, Lund University, Klinikgatan 32, SE-22184 Lund, Sweden; Wallenberg Centre for Molecular Medicine, Lund University, Klinikgatan 32, SE-22184 Lund, Sweden; Department of Physiology, Institute of Theoretical Medicine, Faculty of Medicine, University of Augsburg, Universitätsstr. 2, DE-86159 Augsburg, Germany; Pediatrics, Department of Clinical Sciences Lund, Lund University, Lund, Sweden; Department of Pediatrics, Skåne University Hospital, Lund, Sweden; Pediatrics, Department of Clinical Sciences Lund, Lund University, Lund, Sweden; Department of Pediatrics, Skåne University Hospital, Lund, Sweden; Clinical Physiology, Department of Clinical Sciences Lund, Lund University, Lund, Sweden; Department of Clinical Physiology, Skåne University Hospital, Lund, Sweden; Diagnostic Radiology, Department of Clinical Sciences Lund, Lund University, Lund, Sweden; Department of Radiology, Skåne University Hospital, Lund, Sweden

Preterm-born individuals have an increased risk of developing cardiovascular disease.^1^ Biomarkers identifying individuals at risk are important for early detection and to optimize prevention strategies. Elevated blood pressure (BP) and increased arterial stiffness are both biomarkers of increased cardiovascular risk already in childhood and adolescence after preterm birth.^2^ Since inflammation and cardiovascular disease are intertwined, plasma biomarkers of inflammation evolved as interesting predictors of disease progression and outcome.^3^

The current study explored associations between plasma biomarkers of inflammation, including sphingosine-1-phosphate (S1P) and the cardiovascular biomarkers BP and aortic stiffness with respect to prematurity and sex. As part of a prospective cohort study of adolescents born very preterm (approved by the Regional Ethical Review board in Lund, Sweden; Dnr 2013/244),^4^ this exploratory study included adolescents born before 30 weeks of gestation due to verified foetal growth restriction or with normal foetal growth (*n* = 50; 54% girls) and term-born controls matched for sex (*n* = 29; 52% girls) who accepted to participate in a follow-up study during adolescence (*N* = 79 out of *N* = 102). Adolescent participants voluntarily underwent sampling of blood and urine, 24-h ambulatory BP measurement, and magnetic resonance imaging for thoracic aorta stiffness assessment and S1P concentration determination by liquid chromatography –coupled tandem mass spectrometry.^5^ Group differences between very preterm and term groups were assessed by Mann–Whitney *U* test with exact *P*-value computation. Plasma markers of inflammation quantified at the hospital laboratory or assessed by Bio-Plex Pro™ (Bio-Rad Laboratories, Hercules, CA, USA) were tested for correlation with BP, pulse wave velocity (PWV), and aortic distensibility using Spearman’s rank-order correlation testing. Fisher’s *Z* test was used to assess the significance of differences between two correlation coefficients from independent samples. To control for family-wise error rates for all presented comparisons, additional adjusted *P*-value computation was performed using the Holm method. The investigation conforms to the principles outlined in the Declaration of Helsinki. All participants and when appropriate their guardians provided written informed consent prior to participation.

Preterm birth and a subsequent increase in BP and arterial stiffness are well described^1,2,4^ and confirmed by results in the current study. Additional sex-specific analysis revealed higher BP variables in very preterm boys compared to very preterm-born girls, while PWV was higher in very preterm girls compared to girls born at term (*Table 1A*).

**Table 1:** Study cohort characteristics and S1P associations with cardiovascular and inflammation markers

A. Population characteristics	All				Boys				Girls			
	Very preterm	Term	*P*	*P*_adjusted_	Very preterm	Term	*P*	*P*_adjusted_	Very preterm	Term	*P*	*P*_adjusted_
Peri-/neonatal characteristics	*n = 50*	*n = 29*			*n = 23*	*n = 14*			*n = 27*	*n = 15*		
Gestational age at birth (days)	190 (171–208)	280 (269–285)	**<0**.**0001**		194 (171–208)	280 (269–285)	**<0**.**0001**		188 (172–202)	279 (272–285)	**<0**.**0001**	
Birth weight (g)	830 (395–1790)	3470 (2850–4390)	**<0**.**0001**		950 (570–1790)	3485 (2850–4390)	**<0**.**0001**		795 (395–1414)	3470 (3010–4340)	**<0**.**0001**	
Characteristics in adolescence												
Age (years)	15 (13–16)	15 (13–16)	0.639		15 (13–16)	15 (13–16)	0.745		14(13–16)	14 (13–15)	0.836	
BMI (kg/m^2^)	20 (13–28)	21 (15–25)	0.695		20 (15–28)	22 (18–25)	0.394		20 (13–27)	19 (15–25)	0.705	
BSA (m^2^)	1.6 (1.1–2.1)	1.7 (1.3–2.2)	0.162		1.7 (1.3–2.1)	1.7 (1.3–1.8)	0.526		1.5 (1.1–1.8)	1.5 (1.3–1.8)	0.279	
24-h ABPM												
Daytime	*n = 45*	*n = 23*			*n = 21*	*n = 10*			*n = 24*	*n = 13*		
SBP (mmHg)	122 (104–143)	116 (103–139)	**0**.**020**	**0**.**020**	129 (108–143)^a^	120 (105–139)	0.105	0.199	118 (104–131)	114 (103–123)	0.109	0.109
DBP (mmHg)	73 (63–81)	69 (59–83)	**0**.**012**	**0**.**023**	73 (63–81)	71 (59–83)	0.159	0.159	73 (63–81)	68 (65–77)	**0**.**049**	0.139
MAP (mmHg)	89 (77–101)	84 (76–101)	**0**.**011**	**0**.**032**	92 (77–101)^a^	86 (76–101)	0.087	0.239	87 (79–96)	84 (78–90)	0.058	0.109
Nighttime												
SBP (mmHg)	107 (92–132)	101 (88–120)	**0**.**048**	0.094	108 (98–132)^a^	105 (96–120)	0.147	0.277	105 (92–121)	101 (88–109)	0.179	0.446
DBP (mmHg)	57 (47–70)	55 (50–66)	0.359	0.359	57 (50–66)	59 (51–66)	0.950	0.950	57 (47–70)	54 (50–64)	0.236	0.416
MAP (mmHg)	75 (65–88)	73 (57–84)	**0**.**036**	0.104	77 (67–88)^a^	74 (57–84)	0.079	0.219	75 (65–84)	72 (64–80)	0.263	0.263
Arterial stiffness	*n = 43*	*n = 26*			*n = 22*	*n = 12*			*n = 21*	*n = 14*		
Pulse wave velocity (m/s)	3.7 (3.1–5.1)	3.6 (3.1–5.0)	0.435	0.819	3.7 (3.1–4.8)	3.9 (3.4–5.0)^a^	0.217	0.519	3.7 (3.2–5.1)	3.5 (3.1–4.4)	**0**.**034**	0.098
Distensibility ascending aorta (10^−3^ mmHg^−1^)	9.3 (4.4–14.7)	9.4 (6.0–12.5)	0.941	0.941	8.9 (6.1–13.1)	9.5 (6.0–12.5)	0.511	0.761	10.0 (4.4–14.7)	9.3 (6.2–12.2)	0.414	0.656
Distensibility descending aorta (10^−3^ mmHg^−1^)	11.1 (4.8–17.9)	10.6 (7.0–17.7)	0.892	0.988	10.9 (4.8–17.9)	9.8 (8.0–17.2)	0.845	0.845	11.8 (5.9–17.2)	11.2 (7.0–17.7)	0.606	0.606
Plasma markers												
S1P (nmol/L)	719 (412–1484)	656 (378–1644)	0.737	0.737	760 (480–1484)	733 (380–1644)	0.570	0.570	706 (412–1036)	642 (387–1033)	0.800	0.800
MIP-1β (pg/mL)	8.4 (2.9–43.6)	5.8 (2.2–10.1)	**0**.**002**	**0**.**009**	9.3 (3.0–29.2)	7.1 (2.5–10.0)	**0**.**049**	0.222	7.7 (2.9–43.6)	5.6 (2.2–8.1)	**0**.**011**	**0**.**050**
MCP-1 (pg/mL)	20 (10–60)	15.5 (11.3–38.4)	**0**.**006**	**0**.**023**	18.0 (14.8–59.9)	16.4 (11.7–38.4)	0.170	0.524	20.2 (10.0–37.5)	14.4 (11.3–37.4)	**0**.**019**	0.074
CRP (mg/L)	0.2 (0–6.2)	0.13 (0–3.3)	0.192	0.472	0.44 (0.01–6.24)	0.12 (0–3.28)	0.126	0.332	0.22 (0–3.97)	0.17 (0.06–1.12)	0.776	0.949
eGFR (mL/min per 1.73 m^2^)	94 (68–149)	100 (76–144)	0.277	0.477	86 (68–117)	92 (76–103)	0.136	0.253	102 (76–149)^a^	110 (86–144)^a^	0.454	0.838

B. S1P associations (Spearman)	All			Boys			Girls			Very preterm			Term		
		*n = 60*			*n = 27*			*n = 33*			*n = 19*			*n = 26*	
24-h ABPM	r	*P*	*P*_adjusted_	r	*P*	*P*_adjusted_	*r*	*P*	*P*_adjusted_	*r*	*P*	*P*_adjusted_	*r*	*P*	*P*_adjusted_
24-h SBP (mmHg)	0.323	**0.012**	0.059	0.405	**0.036**	0.104	0.279	0.599	0.834	0.368	**0**.**025**	**0**.**049**	0.279	0.198	0.586
24-h DBP (mmHg)	0.340	**0.008**	**0**.**024**	0.355	0.069	0.133	0.346	0.085	0.234	0.368	**0**.**025**	0.059	0.346	0.106	0.201
24-h MAP (mmHg)	0.329	**0.010**	**0**.**019**	0.347	0.076	0.211	0.354	0.221	0.393	0.380	**0**.**020**	**0**.**025**	0.354	0.097	0.267
Daytime															
SBP (mmHg)	0.341	**0.008**	**0**.**016**	0.435	**0.023**	**0.046**	0.362	0.502	0.876	0.391	**0**.**017**	**0**.**050**	0.362	0.090	0.169
DBP (mmHg)	0.333	**0.009**	**0**.**050**	0.386	**0.047**	0.169	0.471	0.097	0.236	0.326	**0**.**049**	0.096	0.471	**0**.**023**	**0**.**046**
MAP (mmHg)	0.362	**0.005**	**0**.**015**	0.440	**0.022**	0.065	0.523	0.157	0.289	0.346	**0**.**036**	0.071	0.523	**0**.**010**	**0**.**029**
Nighttime															
SBP (mmHg)	0.271	**0.036**	0.104	0.393	**0.042**	0.128	0.137	0.851	0.851	0.328	**0**.**047**	0.092	0.137	0.534	0.899
DBP (mmHg)	0.254	**0.050**	0.097	0.252	0.204	0.366	0.122	0.246	0.432	0.324	**0**.**050**	**0**.**050**	0.122	0.578	0.822
MAP (mmHg)	0.205	0.115	0.115	0.210	0.293	0.293	−0.058	0.420	0.805	0.346	**0**.**036**	0.104	−0.058	0.791	0.791
		*n = 60*			*n = 29*			*n = 31*			*n = 22*			*n = 21*	
Arterial stiffness	r	*P*	P_adjusted_	r	*P*	P_adjusted_	r	*P*	P_adjusted_	r	*P*	P_adjusted_	r	*P*	P_adjusted_
Pulse wave velocity (m/s)	0.177	0.177	0.323	0.119	0.538	0.787	0.112	0.634	0.866	0.231	0.182	0.457	0.112	0.596	0.497
Distensibility ascending aorta (10^−3^ mmHg^−1^)	−0.268	**0.019**	0.054	0.020	0.917	0.917	−0.302	**0**.**005**	**0.015**	−0.216	0.213	0.381	−0.302	0.143	0.379
Distensibility descending aorta (10^−3^ mmHg^−1^)	0.132	0.313	0.313	0.341	0.070	0.195	0.220	0.684	0.684	0.056	0.749	0.749	0.220	0.291	0.291
		*n = 60*			*n = 27*			*n = 33*			*n = 18*			*n = 22*	
Plasma markers	*r*	*P*	*P*_adjusted_	*r*	*P*	*P*_adjusted_	*r*	*P*	*P*_adjusted_	*r*	*P*	*P*_adjusted_	r	*P*	*P*_adjusted_
CRP (mg/L)	0.086	0.483	0.483	0.002	0.990	0.990	−0.185	0.564	0.810	0.261	0.103	0.195	−0.185	0.345	0.719
eGFR (mL/min per 1.73 m^2^)	−0.188	0.127	0.335	−0.057	0.745	0.935	0.073	0.614	0.614	−0.310	**0**.**050**	0.145	0.073	0.718	0.920
MIP-1β (pg/mL)	0.134	0.278	0.479	−0.106	0.537	0.901	0.214	0.117	0.315	0.043	0.793	0.793	0.214	0.274	0.722
MCP-1 (pg/mL)	0.249	**0.041**	0.153	0.126	0.465	0.918	0.06	**0**.**044**	0.167	0.379	**0**.**016**	0.061	0.060	0.763	0.763

Part A: Values presented as median (range). Sample size *n* as indicated in the table is the number of participants who agreed to undergo the respective testing modality (i.e. BP measurements, cardiac MRI acquisition, or blood and urine sampling). *n* for girls’ eGFR (*n* = 32) as no value was obtained for one participant. Statistical significance between very preterm and term groups is given by Mann–Whitney *U* test, exact *P*-value computation, and adjusted for family-wise error rates using the Holm method. P-values of significant comparisons are indicated in bold. All measurements were taken from distinct samples.

^a^Significantly increased compared to opposite sex (*P* < 0.05), within preterm or term groups.

Part B: Values are Spearman correlation coefficients (*r*). All measurements were taken from distinct samples. Sample size *n* as indicated in the table is the number of participants who agreed to undergo the respective testing modality (i.e. BP measurements, cardiac MRI acquisition, or blood and urine sampling). *n* for girls’ eGFR (*n* = 32) as no value was obtained for one participant. Subjects with clinically elevated CRP (>40 mg/L), suggestive of infections, were excluded from statistical analyses of biochemical markers. Some biochemical markers did not return results in the Bio-Plex assay and were therefore excluded from the correlation analyses for the respective marker. Statistical significance is given by Spearman’s rank-order test, exact *P*-value computation, and adjusted for family-wise error rates using the Holm method. P-values of significant comparisons are indicated in bold.

APMP, ambulatory blood pressure measurement; BMI, body mass index; BSA, body surface area; CRP, C-reactive protein; eGFR, estimated glomerular filtration rate; DBP, diastolic blood pressure; MAP, mean arterial pressure; MCP-1, monocyte chemoattractant protein-1; MIP-1β, macrophage inflammatory protein-1β; SBP, systolic blood pressure; S1P, sphingosine-1-phosphate.

Standard clinical inflammation and cardiovascular markers (e.g. C-reactive protein and estimated glomerular filtration rate, eGFR) did not differ between adolescents born very preterm or term, even when stratifying for sex (*Table 1, part A*). However, the observed sex-specific correlation between C-reactive protein and nighttime diastolic BP in only boys (*r*^boys^ = 0.381, *r*^girls^ = 0.214; *z* = 1.859; *P* = 0.032) may be indicative of increased cardiovascular risk in boys as previously discussed.^6^ Similarly, eGFR was higher in girls (*Table 1, part A*) and negatively correlated with daytime diastolic BP in girls (*r*^boys^ = −0.082 and *r*^girls^ = −0.238; *z* = 2.495; *P* = 0.013). These results verify recent findings showing impaired kidney function in adolescent girls born very preterm^7^ and support the strong association between very preterm birth and reduced kidney function.^8^

In addition to established clinical biochemical markers, inflammatory cytokines (including interleukin-2, -7, -8, and -12, interferon gamma, and tumour necrosis factor alpha) were screened to investigate possible associations with BP and arterial stiffness. Most cytokines were detected in low ranges and reported as normal, as expected for generally healthy subjects. However, macrophage inflammatory protein 1 beta (MIP-1β) and monocyte chemoattractant protein-1 (MCP-1) levels were higher in adolescents born very preterm. In girls, MIP-1β further presented with higher levels in the very preterm group compared to term controls (*Table 1, part A*). While MIP-1β did not correlate with BP neither in the entire cohort nor sex-specifically, MCP-1 positively correlated with daytime systolic, diastolic, and mean arterial BP in girls but not in boys (*r*^boys^ = 0.106–0.194; *P* = 0.599–0.332; *P*_adj_ = 0.590–0.422 and *r*^girls^ = 0.355–0.404; *P* = 0.044–0.023; *P*_adj_ = 0.046–0.043). Elevations of the pro-inflammatory cytokines MIP-1β and MCP-1 and higher yet normal BP in the current study are indicative of a possible predisposition for future hypertension. Since higher circulating MCP-1 levels link to higher long-term cardiovascular mortality,^9^ follow-up studies in adulthood may explore the prognostic value of these findings and may potentially help guide future therapy and cardiovascular intervention.

The current study is the first to determine plasma concentration of S1P in adolescents and to investigate its potential association with BP, arterial stiffness, and biochemical markers of inflammation and cardiovascular function in adolescents born very preterm. S1P is an important regulator of both vascular and immune cell function and associates with increments in BP; hence, it has been suggested as a biomarker of vascular dysfunction and a predictive marker for hypertensive disease.^5^ Despite the lack of differences in plasma S1P levels between adolescents born very preterm and term (*Table 1, part A*), S1P correlated with several BP variables (*Table 1B*). This supports the recently proposed associations between plasma S1P and systolic BP in adults.^5^ Sex-specificity of different correlations between S1P and BP variables remained significant only for daytime SBP in boys after correction for multiple comparisons (see *P*_adj_ vs. *P*; *Table 1, part B*).

For most associations with BP, S1P performed similar or better than routine clinical markers, including eGFR (daytime diastolic BP: *r* = −0.366; *P*_adj_ = 0.032 vs. *r* = 0.333; *P*_adj_ = 0.050 for S1P) or CRP (*r* = 0.152–0.112; *P*_adj_ = 0.247–0.778 vs. *r* = 0.362–0.323; *P*_adj_ = 0.044–0.047 for S1P). In contrast to CRP and eGFR, S1P further correlated with aortic distensibility sex-specifically in girls (*Table 1, part B*).

Although associations between S1P and markers of inflammation and cardiovascular disease have been reported,^5^ controversies regarding sex-dependency of S1P plasma responses remain. Considering the effect of oestrogen on S1P plasma concentrations,^10^ there is a need to establish reference values for S1P plasma levels in adolescents. Nonetheless, S1P emerges as promising marker for increased cardiovascular risk as the current study showed a convincing correlation with both BP and aortic distensibility in contrast to classical clinical markers.

In conclusion, S1P and the inflammatory cytokine MCP-1 outperformed standard clinical biochemical markers with respect to their correlations with BP and/or arterial stiffness. Thus, the use of S1P and MCP-1 may potentially help guide cardiovascular risk stratification in those born very preterm.

## Limitations

The sample size may be considered small, but it has been shown to be adequate in previous studies using methods with high accuracy and sensitivity that also limit potential power and type II errors. Additionally, studies in this area that assess cardiovascular parameters in a similar manner (i.e. using cardiac MRI, PWV, ABPMs, and clinical plasma markers) are generally similarly powered. This study is explorative in nature and therefore reports adjusted *P*-values in addition to *P*-values that have not been corrected for multiple comparisons. As a hypothesis-generating study, the objective was not to establish causality between or within observed relationships. Further studies are needed to explore the generalizability and potential clinical practicality of the identified biomarkers. Moreover, verification of S1P and MCP-1 levels and their associations with BP and arterial stiffness is warranted in other relevant cohorts testing the effect of preterm birth on cardiovascular health in adolescents and later in life. The original study included all foetuses who met the inclusion criteria and presented at Skåne University Hospital, Lund, Sweden, between 1998 and 2004.^4^ This demographic represents the general population of the southern part of Sweden. Race and ethnicity data were not collected.

## Authors’ Contributions

Conception: A.M., E.M., D.L., and E.H. Design of the work: A.M., E.M., D.L., and E.H. Data acquisition: J.L., F.M., and E.H. Data analysis: A.M., J.L., F.M., and E.H. Data interpretation: A.M., J.L., and E.H. Manuscript drafting: A.M., J.L., and F.M. Manuscript revision: A.M., J.L., F.M., E.M., D.L., and E.H. Funding acquisition: A.M., D.L., and E.H. All authors had full access to all data in the study. A.M. and E.H. take responsibility for the integrity of data and the accuracy of data analysis. All authors have read and agreed to the published version of the manuscript.

## Data Availability

Data available on request under consideration of privacy restrictions regarding living subjects.
